# The Function of *BBX* Gene Family under Multiple Stresses in *Nicotiana tabacum*

**DOI:** 10.3390/genes13101841

**Published:** 2022-10-12

**Authors:** Kangkang Song, Bin Li, Hui Wu, Yuxue Sha, Liting Qin, Xingyun Chen, Ying Liu, Heng Tang, Long Yang

**Affiliations:** 1 College of Plant Protection and Agricultural Big-Data Research Center, Shandong Agricultural University, Tai’an 271018, China; 2 Institute of Environment and Sustainable Development in Agriculture, Chinese Academy of Agricultural Sciences, Beijing 100081, China; 3 Key Laboratory of Dryland Agriculture, Ministry of Agriculture and Rural Affairs, Beijing 100081, China; 4 AgricultureIsLife, Gembloux Agro-Bio Tech, Liege University, Passage des Déportés 2, 5030 Gembloux, Belgium

**Keywords:** *Nicotiana tabacum*, *BBX* gene family, expression patterns, stress

## Abstract

B-box (BBX) is a zinc finger transcription factor, which is involved in regulating the growth and development of plants and resisting various stresses. In this study, 43 *NtBBX* genes were identified and divided into five subgroups in tobacco. The members in each subgroup had similar characteristics. The promoter region of *NtBBX* genes had cis-acting elements related to light response, hormone regulation and stress response. Transcriptome analysis showed that *NtBBX30* was significantly up-regulated, and *NtBBX12*, *NtBBX13*, *NtBBX16* and *NtBBX17* were significantly down-regulated under abiotic stresses. The *NtBBX* genes also responded to the infection of *Ralstonia solanacearum*. *NtBBX9*, *NtBBX1*, *NtBBX15* and *NtBBX17* showed the greatest response under stresses. The *NtBBX* genes are expressed in various degrees under different tissues. This research will provide a solid foundation for further study of the biological function of *NtBBX* genes in tobacco.

## 1. Introduction

The B-box (BBX) protein, a zinc finger transcription factor with a B-box domain, has attracted much attention due to its multiple biological functions [1]. The BBX proteins have one to two B-box conservative domains at the N-terminal, and some BBX proteins also contain CCT (CONSTANS, CO-like, and TOC1) domains at the C-terminal [2]. The B-box domain is related to the interaction between specific proteins, and the CCT domain plays a regulatory role in gene transcription [3].

The *BBX* gene has been confirmed to be related to stress tolerance and photomorphogenesis in plants. The *BBX* genes can regulate abiotic stress to adapt plants to adverse external environment. In *Arabidopsis*, *AtBBX18* negatively regulated heat tolerance [4]. *CmBBX24* of chrysanthemum was confirmed to be tolerant to low temperature and drought stress [5,6]. *SlBBX17* of tomato was related to heat stress resistance [7], and *SlBBX7*, *SlBBX9* and *SlBBX20* are involved in cold tolerance [8]. Molecular biological methods have demonstrated that the *BBX* genes could enhance the tolerance to salt and drought in transgenic *Arabidopsis* [9,10,11]. In addition, *BBX* genes were also related to biotic stress. The grapevine *BBX* genes can respond to powdery mildew infection [12]. The many functions of *BBX* genes were intrinsically linked to their ability to defend against abiotic stress through different pathways. *MdBBX7*, a target of *MdMIEL1* E3 ligase, could improve drought tolerance in apple [13]. Sweet potato *IbBBX24* could enhance abiotic stress tolerance by activating peroxidase *IbPRX17* transcription to scavenge reactive oxygen species [14]. Some *BBX* genes have been shown to act in hormone signaling pathways [15]. *AtBBX5* was upregulated by ABA under salt, osmotic, and dehydration stresses [16]. *AtBBX18* antagonized hypocotyl elongation inhibition mediated by blue light by increasing the biological activity GA level [17]. *CmBBX24* regulated the flowering time and abiotic stress tolerance by regulating GA biosynthesis [5,6]. *MdBBX37* positively regulated JA-mediated cold stress resistance in apple [18]. *BBX* genes can be induced to express by light and play an important role in photomorphogenesis [19]. *AtBBX30* and *AtBBX31*, acting downstream of HY5, negatively regulated photomorphogenesis in *Arabidopsis* [20]. Under blue light conditions, *OsBBX14* positively regulated rice photomorphogenesis by activating *OsHY5L1* expression [21]. The *AtBBX22IR* and *AtBBX24IR*, *Arabidopsis BBX* alternative splice variants produced by intron retention inhibited hypocotyl elongation by light mediation in *Arabidopsis* [22].

Tobacco is an important economic crop and one of the perfect model plants in scientific research. In recent years, due to global warming and natural disasters, tobacco is suffering from omnipresent threats. As a transcription factor, the *BBX* gene can participate in plant development, hormone signal transduction and the regulation of biotic and abiotic stresses. Therefore, it will be of great significance to study the function of the *BBX* gene family in stress tolerance and development in tobacco. The *BBX* gene family had been identified and analyzed at the whole genome level in many species, including rice [23], wheat [24], tomato [8], petunia [25], apple [26], pear [27] and grape [28]. With the complete sequencing of the whole genome of tobacco, the systematic identification and analysis of the *BBX* gene family become possible. In this study, the physicochemical properties, conservative domains, gene structure, cis-acting elements and gene expression pattern of the tobacco *BBX* gene family was comprehensively analyzed. Phylogenetic analysis can understand the evolutionary relationship of gene families among different species [29,30,31]. Transcriptome analysis has been used to identify potential genes that play an important role in plant development [32,33,34]. This study will provide valuable information for further functional research of *BBX* genes in tobacco and also provide a reference for subsequent molecular mechanism research.

## 2. Materials and Methods

### 2.1. Identification of BBX Genes in the Nicotiana Tabacum L. genome

The tobacco TN90 genome was obtained from the NCBI (https://www.ncbi.nlm.nih.gov/assembly/GCF_000715135.1/ (accessed on 8 May 2021)) [35]. The hidden Markov model (HMM) profile of the B-box domain (Pfam00643) downloaded from the Pfam database (https://pfam.xfam.org/ (accessed on 18 November 2021)) [36] was used as a query to identify *BBX* genes in the proteins sequences file of the tobacco genome using HMMER3.0. When there are multiple transcripts of the same gene, the longest transcript is selected as the *BBX* gene. Proteins sequences and the sequences ID of tobacco *BBX* genes were obtained after deduplication and identification for the B-box conserved domain using the Conserved Domain Database (http://www.ncbi.nlm.nih.gov/Structure/cdd/wrpsb.cgi (accessed on 2 December 2021)) [37]. The number of amino acids (AA), molecular weight (MW), isoelectric point (pI), and grand average of hydropathicity of the corresponding amino acid sequence (GRAVY) were calculated by the ExPASy website (http://web.expasy.org/protparam/ (accessed on 4 December 2021)) using proteins sequences of tobacco *BBX* genes [38]. The subcellular localization (Loc) of tobacco BBX proteins was predicted through WoLF PSORT (https://www.genscript.com/wolf-psort.html (accessed on 4 December 2021)) [39].

### 2.2. Phylogenetic and Conserved Domain Alignments Analysis

The Arabidopsis BBX proteins sequences were attained from the TAIR database (http://www.arabidopsis.org (accessed on 17 September 2021)) [40]. The ClustalW module within MEGA-X was used to align the BBX proteins of tobacco and *Arabidopsis* thaliana, and the phylogenetic tree was constructed using the Neighbor-Joining (NJ) method with 1000 bootstrap-replications [41]. The iTOL website (https://itol.embl.de/ (accessed on 11 June 2022)) was used to beautify the phylogenetic tree [42]. Amino acid sequences of the B-box and CCT conserved domains arranged through CDD results were shown by TBtools [43]. These conserved domains sequences were aligned with ClustalW and GeneDoc software, and sequence logos were generated using Weblogo (http://weblogo.berkeley.edu/logo.cgi (accessed on 6 October 2022)) [44].

### 2.3. Analysis of Gene Structure and Conserved Motifs

The tobacco *BBX* gene structure information was extracted from a genome annotation GFF file. The conserved motif of the tobacco BBX proteins was predicted by the MEME website (https://meme-suite.org/meme/tools/meme (accessed on 6 October 2022)) with ten motifs; the minimum width was set to 6, and the maximum width was set to 50 [45]. The gene structure information was extracted from GFF gene annotation files. The conserved motif and exon–intron structure were drawn using TBtools.

### 2.4. Cis-Acting Elements Analysis in the BBX Genes Promoter

The promoters sequences were considered to be 2000 bp upstream of the first CDS of the tobacco *BBX* gene and was gained from the genome file and genome annotation file by TBtools. The cis-acting element was predicted using the PlantCARE website (http://bioinformatics.psb.ugent.be/webtools/plantcare/html/ (accessed on 21 November 2021)) [46] and was visualized by TBtools.

### 2.5. Expression Analysis of BBX Genes in Different Stress, Hormone and Tissue

RNA-seq data were used to analyze the expression patterns of *BBX* genes in tobacco under different conditions. RNA-seq data including NaCl (SRP193166), NaHCO_3_ (SRP193166), Cold (SRP097876), dehydration (SRP301492), *Ralstonia solanacearum* (*R. solanacearum*) (SRP336664), Melatonin (SRP301492), and different plant tissues (SRP101432) were obtained from the Sequence Read Archive (SRA) database (https://www.ncbi.nlm.nih.gov/sra/ (accessed on 10 May 2022)) [47]. The data of stems, stem apexes and roots were downloaded to analyze the difference of gene expression between different plant tissues. In addition, transcriptome data under abscisic acid (ABA) treatment came from laboratory data [48].

Trimmomatic [49] was used to remove the adapter and cut off the first 12 bases of reads (except ABA). Hisat2 [50] was exploited to establish the genome index and map reads. The Samtools [51] command was applied to convert the sam file to a bam file. Stringtie [52] was employed to calculate FPKM. The counts value was obtained by the prepDE.py3 program provided by stringtie. The differently expressed genes were analyzed by using DEseq2 [53]. The differently expressed genes screening standard was |log2FoldChange|≥ 1 and padj ≤ 0.05. The results were displayed by TBtools with a heatmap.

## 3. Results

### 3.1. Identification of BBX Genes in the Tobacco Genome

To identify *BBX* genes in the tobacco genome, the hidden Markov model (HMM) profile of the B-box domain (Pfam00643) was employed to search protein sequence files. In total, 43 tobacco BBX proteins sequences were obtained after deleting repeated sequences and identifying conserved domains. For the consistency of naming, tobacco *BBX* genes were named *NtBBX1* to *NtBBX43* (Table 1). In addition, multiple transcripts were found for four *NtBBX* genes (Table S1). The length of the NtBBX proteins sequences ranged from 176 amino acids to 473 amino acids. The maximum molecular weight and the minimum molecular weight were 51,997.34 and 19,539.31, respectively. Their pI ranged from 4.31 to 8.42 and grand average of hydropathicity (GRAVY) scores ranged from −0.938 to −0.282. Subcellular localization showed that most (79%) NtBBX proteins existed in the nucleus, such as NtBBX1, NtBBX2, and NtBBX8.

### 3.2. Conserved Domain and Phylogenetic Analysis of NtBBX Gene Family

**Figure 1:**
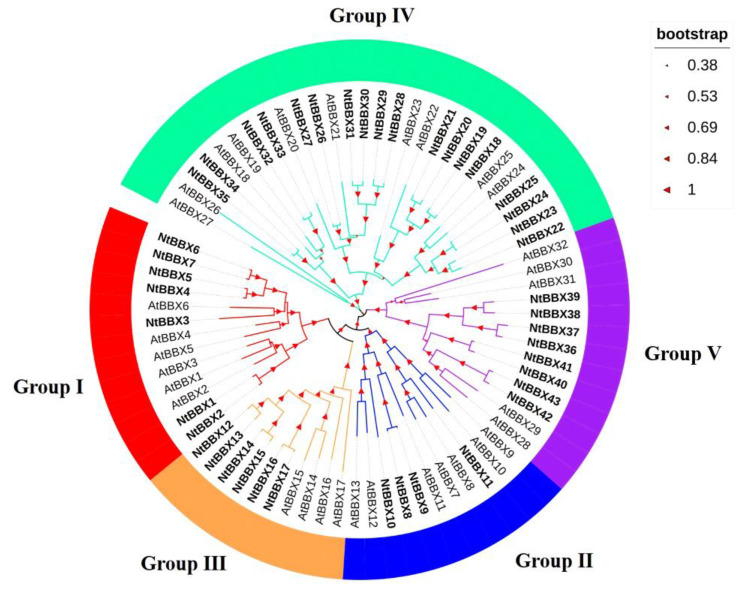
Phylogenetic tree of BBX proteins sequences from tobacco and Arabidopsis. The phylogenetic tree was constructed using MEGA-X with the Neighbor-Joining (NJ) method with 1000 bootstrap replications. The tree was divided into five groups, and the bootstrap values were indicated by the size of asterisks.

**Figure 2:**
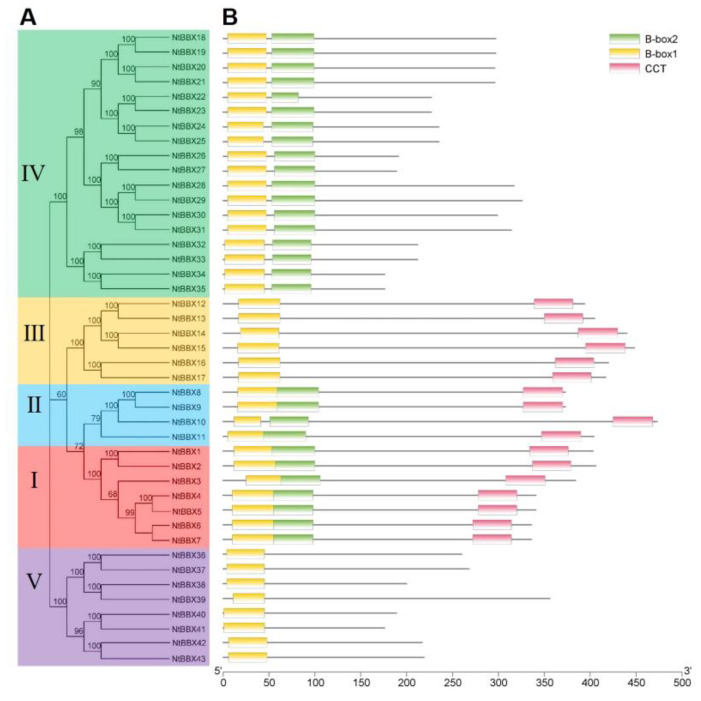
Conserved domains of NtBBX proteins. (**A**) Phylogenetic tree of NtBBX proteins sequences from tobacco. The phylogenetic tree was constructed using MEGA-X with the Neighbor-Joining (NJ) method with 1000 bootstrap replications; (**B**) Conserved domains of NtBBX. B-box1 domains are indicated by yellow boxes, B-box2 domains are indicated by green boxes, and CCT domains are indicated by pink boxes.

**Figure 3:**
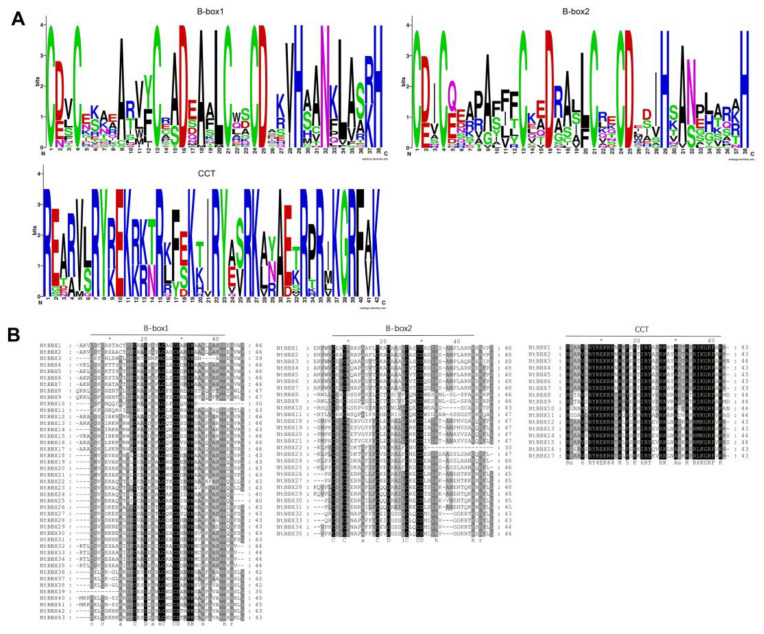
Sequence characteristics of domains of NtBBX proteins. (**A**) Sequence logos alignment of the B-box1, B-box2, and CCT domains. Each colored letter represents an amino acid residue. The height of amino acids is directly proportional to the conservation of amino acids in this position; (**B**) Multi-sequence alignment of the B-box1, B-box2 and CCT domains of the NtBBX proteins. The black background means that the amino acids here are absolutely conservative.

In order to study the phylogenetic relationships of the *NtBBX* family, a phylogenetic tree was constructed using 43 tobacco BBX proteins and 32 *Arabidopsis* BBX proteins (Figure 1). According to the phylogenetic analysis, 43 *NtBBX* genes were divided into five subgroups. The number of members in the five subgroups was 7, 4, 6, 18, and 8, respectively. Each member of subgroup I and subgroup II had both contained two B-box domains and one CCT domain (B1+B2+CCT) (Figure 2), but they had differences in amino acid sequence (Figure 3A). Each member of subgroup III contained a B-box domain at the amino terminus and a CCT domain at the carboxy terminus. Each member of subgroup IV and subgroup V had only the B-box domain. The difference was that each member of subgroup IV had two B-box domains, and each member of subgroup V had one B-box domain.

### 3.3. Domain Alignments and Sequence Logos

In order to explore the amino acid sequence composition of each characteristic domain and its conservation, sequence logos (Figure 3A), and multi-sequence alignment (Figure 3B) of domains were performed. The results showed that B-box1 and B-box2 had high sequence similarity. In contrast to the B-box2 domain, the B-box1 domain was more conservative for the Alanine (A) of 9, 15, 18, and 35 of B-box1. In addition, the CCT domain was also highly conservative, such as Arginine (R), Lysine (K), and Tyrosine (Y).

### 3.4. Gene Structure and Conserved Motifs

**Figure 4:**
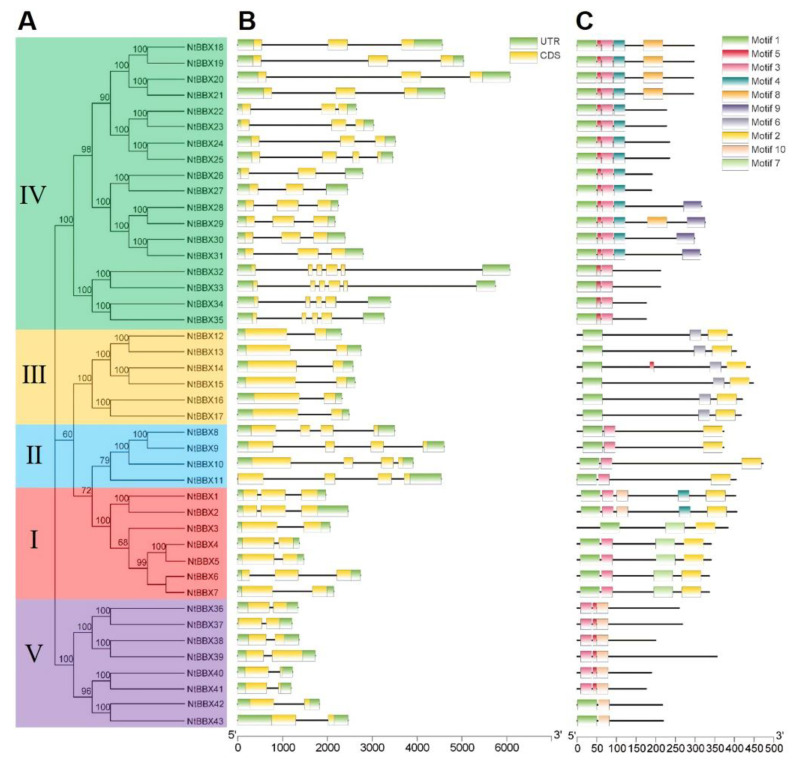
Gene structure and motif of *NtBBX* gene family. (**A**) Phylogenetic tree of NtBBX proteins sequences from tobacco; (**B**) Gene structure of *NtBBX* genes. UTR are represented by green boxes, CDS are represented by yellow boxes, and black lines represent intron; (**C**) Motif of NtBBX proteins. Ten different categories of motif are represented by boxes of ten colors. Detailed sequence features of motifs are shown in Supplementary Figure S1.

The number of exons of *NtBBX* genes ranged from 2 to 6. In the same subgroup, *NtBBX* genes had similar numbers of exons (Figure 4B). Except for *NtBBX1*, *NtBBX2* and *NtBBX6* in subgroup I, had three exons, and the rest had two exons.

All members of the subgroups II, III, and V had four, two, and two exons, respectively. Most members of the subgroup IV had three exons. Gene members of the same subgroup had similar motif types (Figure 4C).

### 3.5. Cis-Acting Elements of NtBBX Genes

**Figure 5:**
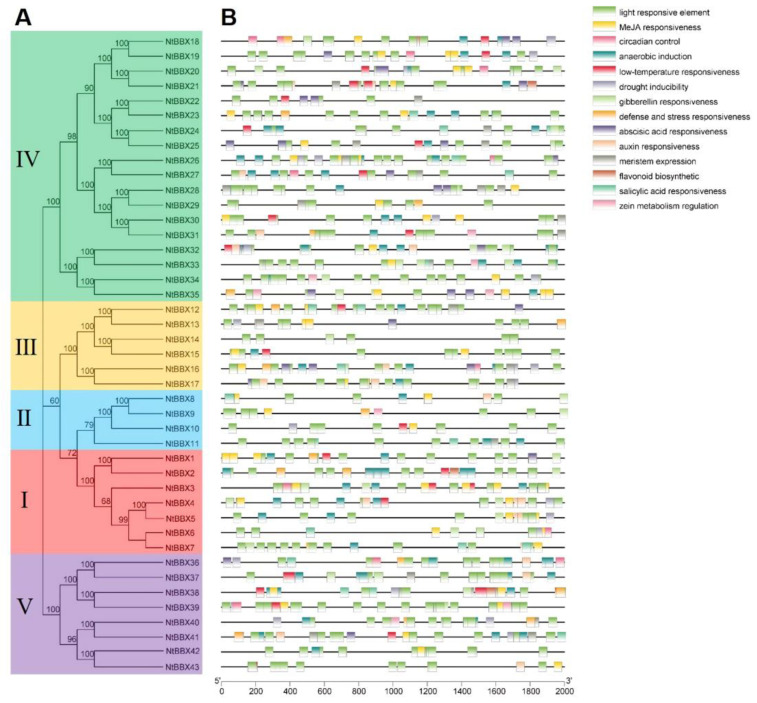
Cis-acting elements in promoter region of *NtBBX* genes. (**A**) Phylogenetic tree of NtBBX proteins sequences from tobacco; (**B**) Cis-acting elements. Detailed information of cis-acting elements is provided in Supplementary Table S2.

The cis-acting elements of the promoter region of *NtBBX* genes were analyzed to reveal the potential function of the *NtBBX* family (Figure 5). Three main types of cis-acting elements in the *NtBBX* genes were light-responsive elements, hormone-responsive elements and stress-responsive elements. The light-responsive elements were widely distributed in each *NtBBX* gene. The MeJA responsiveness element, gibberellin responsiveness element, auxin responsiveness element, abscisic acid responsiveness element, and salicylic acid responsiveness element belonged to hormone-responsive elements. The anaerobic induction element, low-temperature responsiveness element, drought inducibility element and defense and stress responsiveness element belonged to abiotic stress-responsive elements. In addition to the three kinds of elements mentioned above, *NtBBX* genes contained elements related to circadian control, meristem expression, flavonoid biosynthetic, and zein metabolism regulation.

### 3.6. Expression Patterns of NtBBX Genes under Abiotic Stress

**Figure 6:**
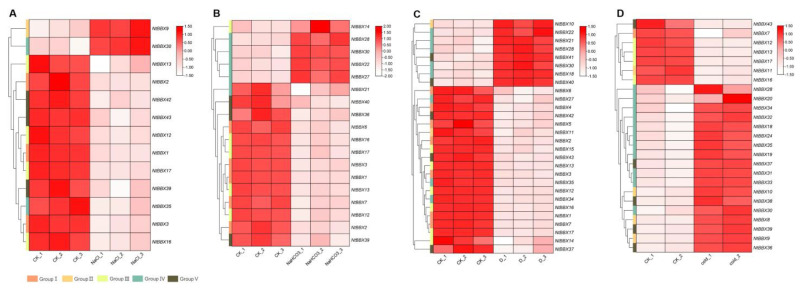
Expression patterns of *NtBBX* genes under abiotic stress. (**A**) Expression pattern of *NtBBX* genes under NaCl stress; (**B**) Expression pattern of *NtBBX* genes under NaHCO_3_ stress; (**C**) Expression pattern of *NtBBX* genes under dehydration stress; (**D**) Expression pattern of *NtBBX* genes under cold stress. Subgroups I–V are represented by different colored boxes.

Under salt stress, 13 *NtBBX* genes were differentially expressed (Figure 6A). The expression levels of *NtBBX9* and *NtBBX30* were significantly up-regulated, while the other *NtBBX* genes were significantly down-regulated. When tobacco was exposed to alkali stress, the expression levels of 18 *NtBBX* genes were significantly changed (Figure 6B). Five *NtBBX* genes were significantly up-regulated, which were *NtBBX14*, *NtBBX17*, *NtBBX22*, *NtBBX27*, and *NtBBX30*. Under dehydration stress, the expression levels of 28 *NtBBX* genes were changed significantly (Figure 6C). Twenty *NtBBX* genes were significantly down-regulated, and eight *NtBBX* genes were significantly up-regulated. Interestingly, all *NtBBX* genes of subgroup I and subgroup III were significantly down-regulated. The expression of 25 *NtBBX* genes was significantly different between cold stress and normal condition (Figure 6D). The majority (11/18) of the up-regulated *NtBBX* genes belonged to subgroup IV, and the majority (4/7) of the down-regulated *NtBBX* genes belonged to subgroup III.

### 3.7. Expression Patterns of NtBBX Genes under Biotic Stress

**Figure 7:**
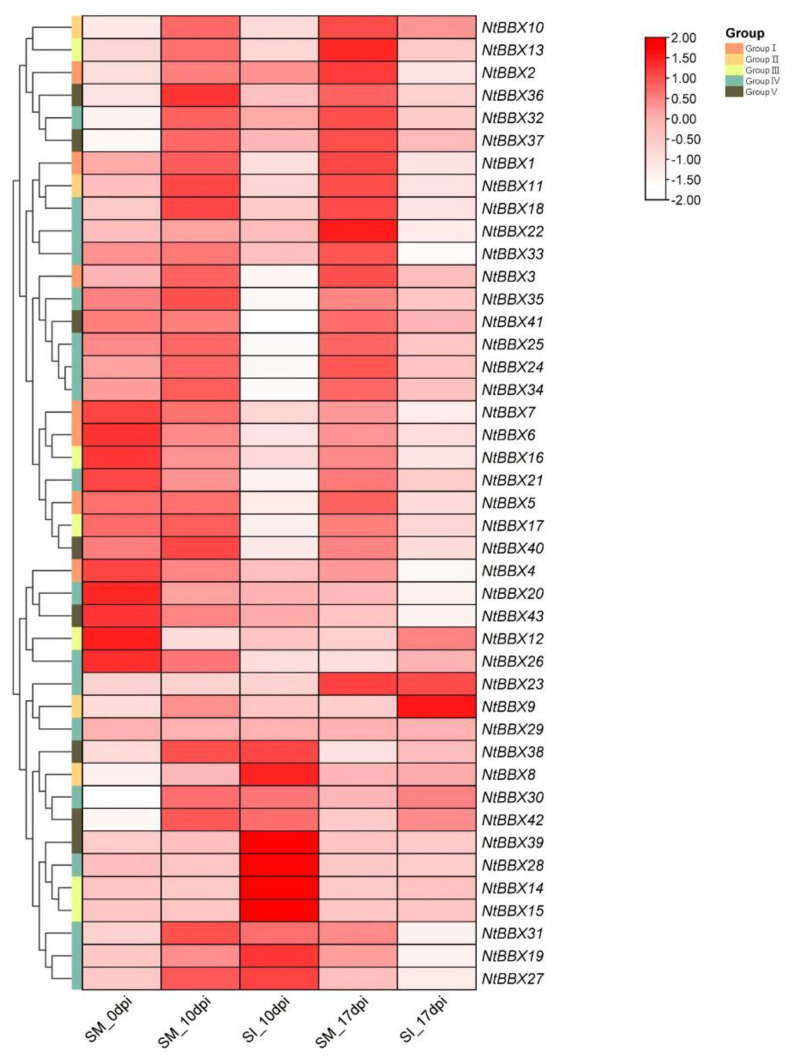
Expression patterns of *NtBBX* genes under biotic stress. SI, no infection. SM, infection with *R. solanacearum*. Dpi, days after infection. Subgroups I–V are represented by different colored boxes.

After tobacco was infected by *R. solanacearum*, different *NtBBX* genes showed different expression patterns (Figure 7). The expression levels of some *NtBBX* genes were significantly increased after 10 days of infection with *R. solanacearum*, including *NtBBX8*, *NtBBX14*-*15* and *NtBBX28*. The expression levels of some *NtBBX* genes were significantly decreased after 10 days, including *NtBBX17*, *NtBBX21*, *NtBBX35* and *NtBBX40-41*. The expression level of *NtBBX9* was significantly increased after 17 days of infection with *R. solanacearum*. The expression levels of some *NtBBX* genes were significantly decreased after 17 days, including *NtBBX2*, *NtBBX16* and *NtBBX22-23*. After 10 and 17 days, the expression levels of some *NtBBX* genes were significantly decreased, including *NtBBX1*, *NtBBX24-25* and *NtBBX34*.

### 3.8. Expression Patterns of NtBBX Genes under Hormone Treatment

**Figure 8:**
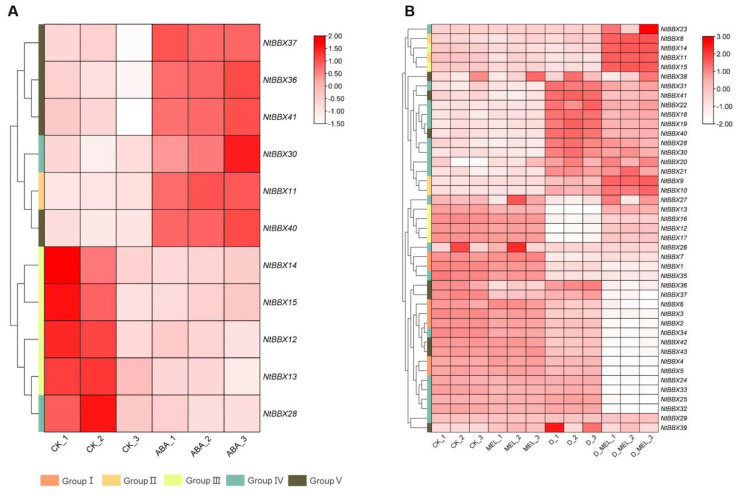
Expression of *NtBBX* genes under hormone induction. (**A**) Expression pattern of *NtBBX* genes under ABA treatment; (**B**) Expression pattern of *NtBBX* genes under dehydrate stress and melatonin treatment. D, dehydrate. MEL, melatonin. Subgroups I–V are represented by different colored boxes.

Eleven *NtBBX* genes were differentially expressed under ABA treatment (Figure 8A). Six *NtBBX* genes were significantly up-regulated, which were *NtBBX11*, *NtBBX30*, *NtBBX36–37* and *NtBBX40–41*. Five *NtBBX* genes were significantly down-regulated, which were *NtBBX12–15* and *NtBBX28*.

The *NtBBX* genes did not respond strongly under a single melatonin treatment, and the differentially expressed genes had only *NtBBX15*, *NtBBX36,* and *NtBBX37* (Figure 8B). Under dehydration stress and melatonin treatment, 21 *NtBBX* genes were significantly down-regulated, including *NtBBX1–7*, *NtBBX12–13*, *NtBBX16–17*, *NtBBX24–25*, *NtBBX32–37* and *NtBBX42–43*. Nine *NtBBX* genes were significantly up-regulated, including *NtBBX9–11*, *NtBBX14–15*, *NtBBX21–22*, *NtBBX30* and *NtBBX40*.

### 3.9. Expression Patterns of NtBBX Genes in Different Tissues

**Figure 9:**
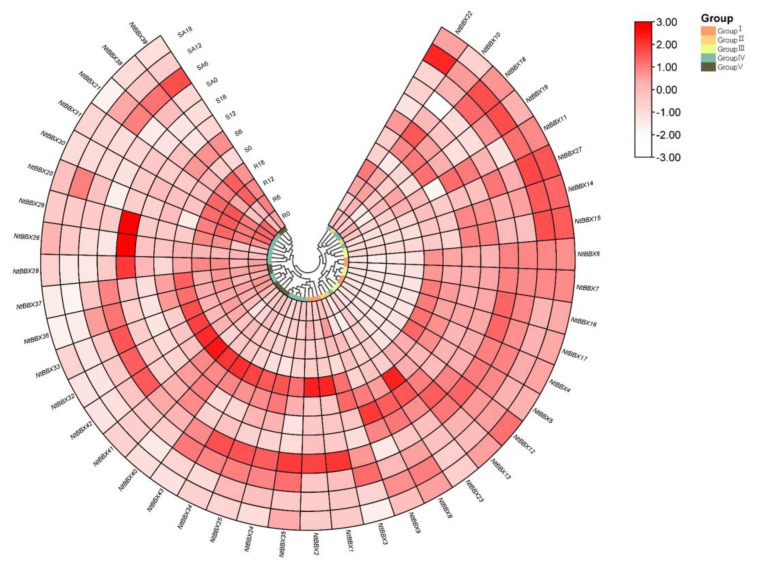
Expression patterns of *NtBBX* genes in different tissues. R, roots. S, stems. SA, stem apexes. Subgroups I–V are represented by different colored boxes.

*NtBBX* genes were expressed in all three tissues (Figure 9). *NtBBX* genes were mainly expressed in stems and stem apexes, but less in roots, such as *NtBBX4*, *NtBBX5*, *NtBBX6*, *NtBBX7*, *NtBBX16* and *NtBBX17*. *NtBBX* genes with high expression in stems mainly belonged to subgroup I and subgroup IV. Some *NtBBX* genes were expressed more in stem apexes than in stems, for example, *NtBBX6*, *NtBBX7* and *NtBBX35*.

## 4. Discussion

BBX is a transcription factor of zinc finger protein, which plays a prominent role in plant growth and development, hormone response, and stress tolerance [54], but the function of *BBX* genes in tobacco has not been studied. In this study, the phylogeny, gene structure, cis-acting element, and expression pattern under diverse conditions of the *NtBBX* genes were comprehensively analyzed in tobacco. Genome-wide analysis of the *NtBBX* genes will establish a solid foundation for *NtBBX* genes function research in tobacco.

### 4.1. The Number and Classification of Tobacco NtBBX Genes

In this study, 43 *NtBBX* genes were obtained from the tobacco genome data. Even though both tobacco and tomato belong to Solanaceae, the number of *BBX* genes in tobacco is more than that in tomato (29) [8]. It might be that tobacco is an allotetraploid which is a combination of two diploid wild species and has more homologous genes [55]. Whole genome duplication (WGD) and segmental duplication may be important reasons for *NtBBX* gene family expansion [56,57]. The evolutionary tree was constructed using the *BBX* genes of *Arabidopsis thaliana* and tobacco, and finally, the *NtBBX* genes were divided into five subgroups. This was consistent with the grouping of *Arabidopsis* [2]. Coincidentally, the type and number of conserved domains in the *AtBBX* genes and the *NtBBX* genes were identical in each subgroup. For example, the *AtBBX* genes and the *NtBBX* genes in subgroup III both contain a B-box domain and a CCT domain. The *BBX* genes of tobacco and *Arabidopsis* in the same branch may have similar biological functions.

### 4.2. Tobacco NtBBX Genes and Photomorphogenesis

*BBX* genes are related to the development of light morphology in plants [58]. Cis-acting element analysis showed that a large number of photoresponsive elements were in the promoter region of *NtBBX* genes, for example, G-box, GT1-motif, and TCT-motif. The type and number of cis-acting elements in the promoter region are closely related to the gene’s function, leading to differential expression of the gene [59]. Studies on *Arabidopsis* have shown that *AtBBX21* and *AtBBX22* promote photomorphogenesis, while *AtBBX24* and *AtBBX25* inhibit photomorphogenesis [1,15,19]. From the evolutionary tree, *NtBBX18*–*NtBBX35* were grouped into the same group as the four *Arabidopsis BBX* genes mentioned above, suggesting that they might be involved in the photomorphogenesis of tobacco. The *NtBBX* genes function in the photomorphogenesis will require further study in the future, which is very significant for tobacco improvement.

### 4.3. Tobacco NtBBX Genes and External Stresses

**Figure 10:**
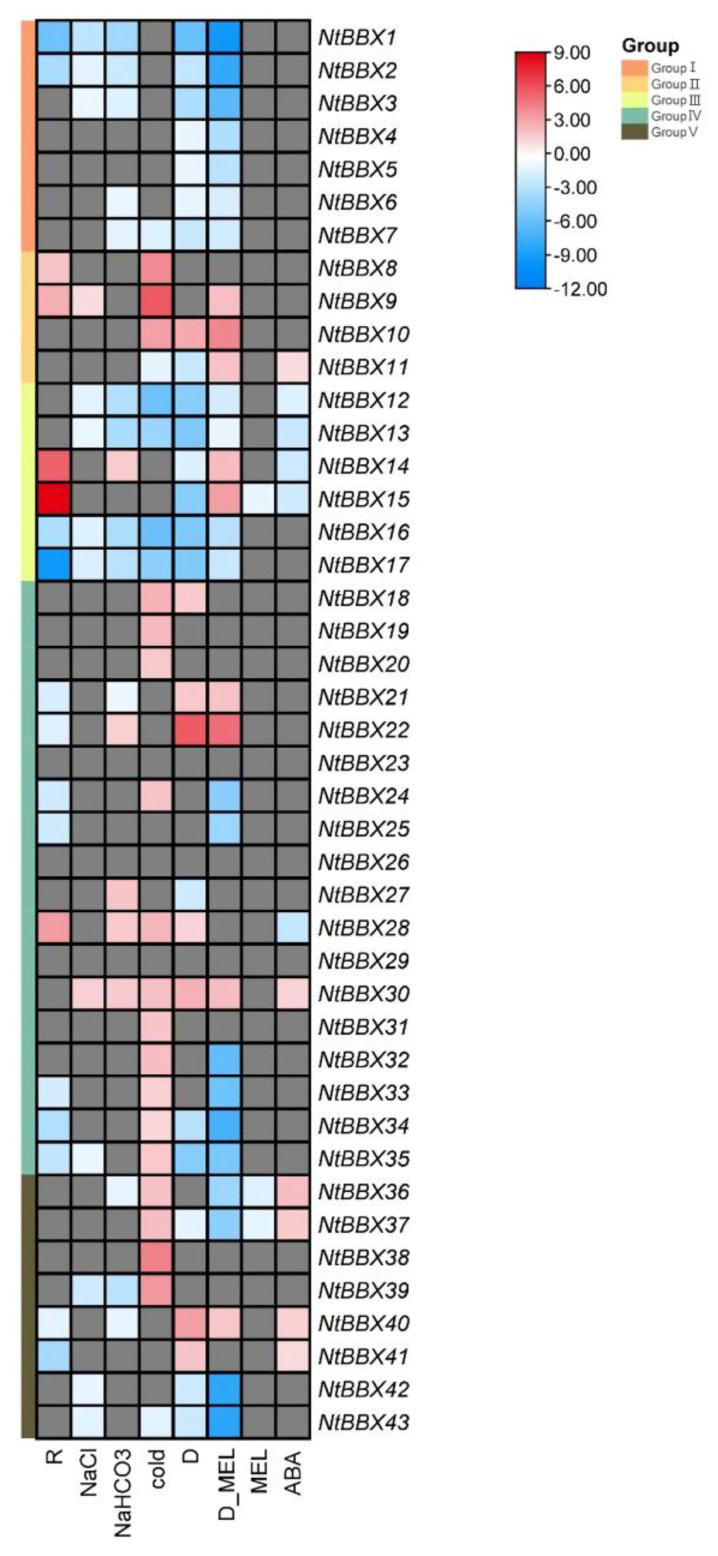
Differential expression under multiple stresses and hormone treatment. The red boxes, blue boxes and gray boxes represent significant up-regulation, significant down-regulation and no significant change for *NtBBX* genes under corresponding conditions, respectively. The color depth of red boxes and blue boxes represents the size of log_2_ fold change.

Stress cis-acting elements were widely present in the promoter region of tobacco *NtBBX* genes, suggesting that tobacco *NtBBX* genes may take part in tobacco response to stress. Therefore, the expression patterns of tobacco *NtBBX* genes were analyzed under NaCl, NaHCO_3_, cold, dehydration, and *R. solanacearum* stresses. The results showed that most tobacco *NtBBX* genes were differentially expressed under five stresses, and some genes distributed in the same subgroup had similar expression patterns (Figure 10). All *NtBBX* genes in subgroup I negatively regulated one or more stresses. All *NtBBX* genes in subgroup III had negative regulatory effects on dehydration stress. In four abiotic stresses, the number of *NtBBX* genes up-regulated by cold stress was the largest, which was followed by that by dehydration and NaHCO_3_ stress, and the number of *NtBBX* genes up-regulated by NaCl stress was the smallest. This may suggest that *NtBBX* genes play a more positive role in regulating temperature stress than osmotic stress [60]. Notably, most of the genes significantly up-regulated under these abiotic stresses conditions belong to subgroup IV. A study showed that the tomato subgroup IV *SIBBX20* gene could induce the expression of cold-responsive (COR) genes to adapt to cold stress [8]; the study on *Ginkgo biloba* showed that the salt tolerance of transgenic poplar overexpressing *GbBBX25* was improved [61]. These facts indicate that the fourth subfamily genes have an important effect in plants response to abiotic stress. *NtBBX30* was significantly up-regulated, and *NtBBX12*, *NtBBX13*, *NtBBX16* and *NtBBX17* were significantly down-regulated under the four abiotic stresses. These genes are widely involved in the stress resistance process of tobacco and have an important influence on the growth and development of tobacco, which is worthy of further study on their functions. *NtBBX9 and NtBBX1* showed the greatest response under abiotic stress. Tobacco *NtBBX* genes can respond to *R. solanacearum* infection in different degrees. The expression levels of *NtBBX* genes significantly differentially expressed under abiotic stress also changed under pathogen infection. *NtBBX15* and *NtBBX17* showed the greatest response under *R. solanacearum* stress. As a transcription factor, *NtBBX* genes were closely related to the abiotic and biotic stress tolerance of tobacco. These *NtBBX* genes could be used as the key genes of tobacco resistance to a variety of stresses, and it was expected to create tobacco multi-resistant germplasm by transgenic technology in the late stage. The *NtBBX* genes were highly expressed in stems and stem apexes, but they were relatively low in roots, which indicates that the expression of tobacco *NtBBX* genes was tissue-specific.

### 4.4. Tobacco NtBBX Genes and Hormone Response

A large number of hormone-responsive elements were also present in tobacco *NtBBX* genes promoted such as ABA, GA and SA, suggesting that these genes may regulate many hormone signal transduction pathways and regulate the growth and development processes of tobacco. Transcriptome analysis showed that the tobacco *NtBBX* genes were regulated by ABA and melatonin (Figure 10).

Among the eleven differentially expressed genes under ABA treatment, more than half (4/6) of the up-regulated *NtBBX* genes were located in subgroup V, and more than half (4/5) of the down-regulated *NtBBX* genes were located in subgroup III. Subgroup preference suggests that two groups of *NtBBX* genes may defend against external stress through different signal transduction pathways. The *CmBBX19* of chrysanthemum negatively regulated the drought tolerance through the ABA-dependent pathway [62]. The tobacco *NtBBX* genes may also regulate various abiotic stresses through the ABA pathway. *NtBBX9*, *NtBBX11*, *NtBBX14* and *NtBBX15* were significantly up-regulated under melatonin treatment and dehydration stress, which were contrary to the expression pattern under single dehydration stress, suggesting that these tobacco *NtBBX* genes participated in tobacco resistance to dehydration stress through the melatonin pathway.

ABA and melatonin regulate tobacco *NtBBX* gene to adapt to abiotic stress. The molecular mechanism of the hormone response of *BBX* genes in tobacco and the downstream pathway involved may be complex, which needs to be further explored using physiology and biochemistry combined with forward and reverse genetics.

## 5. Conclusions

In this study, the genome-wide analysis of the tobacco *BBX* gene family was performed, and we identified a total of 43 *NtBBX* genes. The physicochemical properties, phylogeny, conservative domain, gene structure, conservative motif, cis-acting element and expression patterns under various conditions of the *NtBBX* gene family were systematically analyzed in tobacco. A large number of light-responsive elements, hormone responsive elements, and stress-responsive elements existed in tobacco *NtBBX* genes. Transcriptome analysis showed that the *NtBBX* genes were responsive to salt, alkali, cold, dehydration and *R. solanacearum* infection. *NtBBX9*, *NtBBX1*, *NtBBX15* and *NtBBX17* showed the greatest response under stress. *NtBBX30* expression was significantly up-regulated in all four abiotic stresses, and *NtBBX12*, *NtBBX13*, *NtBBX16*, and *NtBBX17* were significantly down-regulated. The *NtBBX* genes of subgroup IV may have an essential impact on cold stress. The *NtBBX* genes also showed tissue specificity, with more expression in stems and stem apexes but less in roots. In conclusion, these studies will establish a solid foundation for further research on *NtBBX* gene function in tobacco.

## Figures and Tables

**Table 1:** The fundamental information of *NtBBX* genes.

Gene Name	Gene ID	Transcript	AA	MW	pI	GRAVY	Loc
*NtBBX1*	*LOC107765723*	XM_016584401.1	403	44,755.71	5.52	−0.644	nucl
*NtBBX2*	*LOC107785832*	XM_016607219.1	406	44,973.90	5.78	−0.665	nucl
*NtBBX3*	*LOC107761869*	XM_016580159.1	384	42,380.80	6.42	−0.301	extr
*NtBBX4*	*LOC107786858*	XM_016608359.1	341	37,974.51	5.83	−0.509	chlo
*NtBBX5*	*LOC107799876*	XM_016623011.1	341	37,860.29	5.72	−0.484	chlo
*NtBBX6*	*LOC107814243*	XM_016639620.1	336	37,476.91	5.77	−0.542	chlo
*NtBBX7*	*LOC107826614*	XM_016653614.1	336	37,442.90	5.96	−0.532	chlo
*NtBBX8*	*LOC107814991*	XM_016640497.1	373	41,794.43	5.3	−0.66	nucl
*NtBBX9*	*LOC107827338*	XM_016654437.1	373	41,764.53	5.37	−0.655	nucl
*NtBBX10*	*LOC107818548*	XM_016644579.1	473	51,997.34	7.79	−0.663	nucl
*NtBBX11*	*LOC107771275*	XM_016590616.1	404	44,346.24	5.04	−0.526	nucl
*NtBBX12*	*LOC107778231*	XM_016598444.1	394	45,011.52	5.94	−0.8	nucl
*NtBBX13*	*LOC107814123*	XM_016639456.1	405	45,959.53	5.21	−0.735	nucl
*NtBBX14*	*LOC107786610*	XM_016608118.1	440	50,204.22	5.08	−0.795	nucl
*NtBBX15*	*LOC107825347*	XM_016652196.1	448	51,001.99	5.16	−0.814	nucl
*NtBBX16*	*LOC107761249*	XM_016579455.1	420	47,672.21	5.07	−0.755	nucl
*NtBBX17*	*LOC107799644*	XM_016622785.1	417	47,266.65	5.07	−0.789	nucl
*NtBBX18*	*LOC107783851*	XM_016604881.1	297	32,036.76	5.07	−0.394	nucl
*NtBBX19*	*LOC107803806*	XM_016627591.1	297	32,148.86	5.01	−0.426	nucl
*NtBBX20*	*LOC107799951*	XM_016623094.1	296	32,005.84	5.01	−0.283	nucl
*NtBBX21*	*LOC107825567*	XM_016652435.1	296	32,118.16	5.3	−0.282	nucl
*NtBBX22*	*LOC107793240*	XM_016615553.1	227	25,286.95	6.38	−0.307	nucl
*NtBBX23*	*LOC107828748*	XM_016656120.1	227	25,356.01	6.17	−0.293	nucl
*NtBBX24*	*LOC107817419*	XM_016643247.1	235	26,084.50	4.89	−0.391	nucl
*NtBBX25*	*LOC107818998*	XM_016645077.1	235	26,051.52	4.9	−0.38	nucl
*NtBBX26*	*LOC107797652*	XM_016620558.1	191	21,318.80	5.97	−0.458	nucl
*NtBBX27*	*LOC107805058*	XM_016629035.1	189	21,121.60	6.12	−0.452	nucl
*NtBBX28*	*LOC107782278*	XM_016603147.1	317	35,302.56	6.7	−0.502	nucl
*NtBBX29*	*LOC107786262*	XM_016607710.1	326	36,308.76	6.7	−0.498	nucl
*NtBBX30*	*LOC107818039*	XM_016643950.1	299	33,073.23	7.54	−0.445	nucl
*NtBBX31*	*LOC107830932*	XM_016658627.1	314	34,491.80	8.42	−0.438	nucl
*NtBBX32*	*LOC107771491*	XM_016590865.1	212	23,517.62	5.66	−0.53	nucl
*NtBBX33*	*LOC107797424*	XM_016620324.1	212	23,510.67	5.81	−0.554	nucl
*NtBBX34*	*LOC107807066*	XM_016631354.1	176	19,658.42	7.01	−0.588	cyto
*NtBBX35*	*LOC107829475*	XM_016656924.1	176	19,539.31	7.05	−0.535	cyto
*NtBBX36*	*LOC107762308*	XM_016580655.1	260	28,717.17	4.54	−0.83	chlo
*NtBBX37*	*LOC107795590*	XM_016618260.1	268	29,427.73	4.49	−0.882	nucl
*NtBBX38*	*LOC107767469*	XM_016586479.1	200	21,976.38	5.44	−0.755	chlo
*NtBBX39*	*LOC107832542*	XM_016660406.1	356	39,153.73	6.58	−0.737	nucl
*NtBBX40*	*LOC107763696*	XM_016582192.1	189	21,002.61	4.84	−0.938	nucl
*NtBBX41*	*LOC107823765*	XM_016650470.1	176	19,559.18	5.08	−0.898	nucl
*NtBBX42*	*LOC107813335*	XM_016638592.1	217	23,795.63	4.31	−0.836	nucl
*NtBBX43*	*LOC107820983*	XM_016647357.1	219	24,151.06	4.34	−0.843	nucl

Instruction: AA amino acid, MW molecular weight, pI theoretical isoelectric point, GRAVY grand average of hydropathicity, Loc subcellular location. Nucl nucleus, Chlo chloroplast, Cyto cytosol, Extr extracellular.

## Data Availability

Data are contained within the article or Supplementary Material.

## Supplementary Materials

The following supporting information can be downloaded at: https://www.mdpi.com/article/10.3390/genes13101841/s1, Figure S1: Detailed sequence features of ten motifs; Table S1: Multiple transcripts for four *NtBBX* genes. Table S2: Detailed information of cis-acting elements.
