# Supplementary material for: The Function of BBX Gene Family under Multiple Stresses in Nicotiana tabacum
Source: Genes (Basel). 2022 Oct 12;13(10):1841. doi: 10.3390/genes13101841 (PMC9602306; doi:10.3390/genes13101841)
Supplement: Supplementary file 1 [file genes-13-01841-s001.zip › FigureS1ú║Detailed sequence features of ten motifs.pdf]

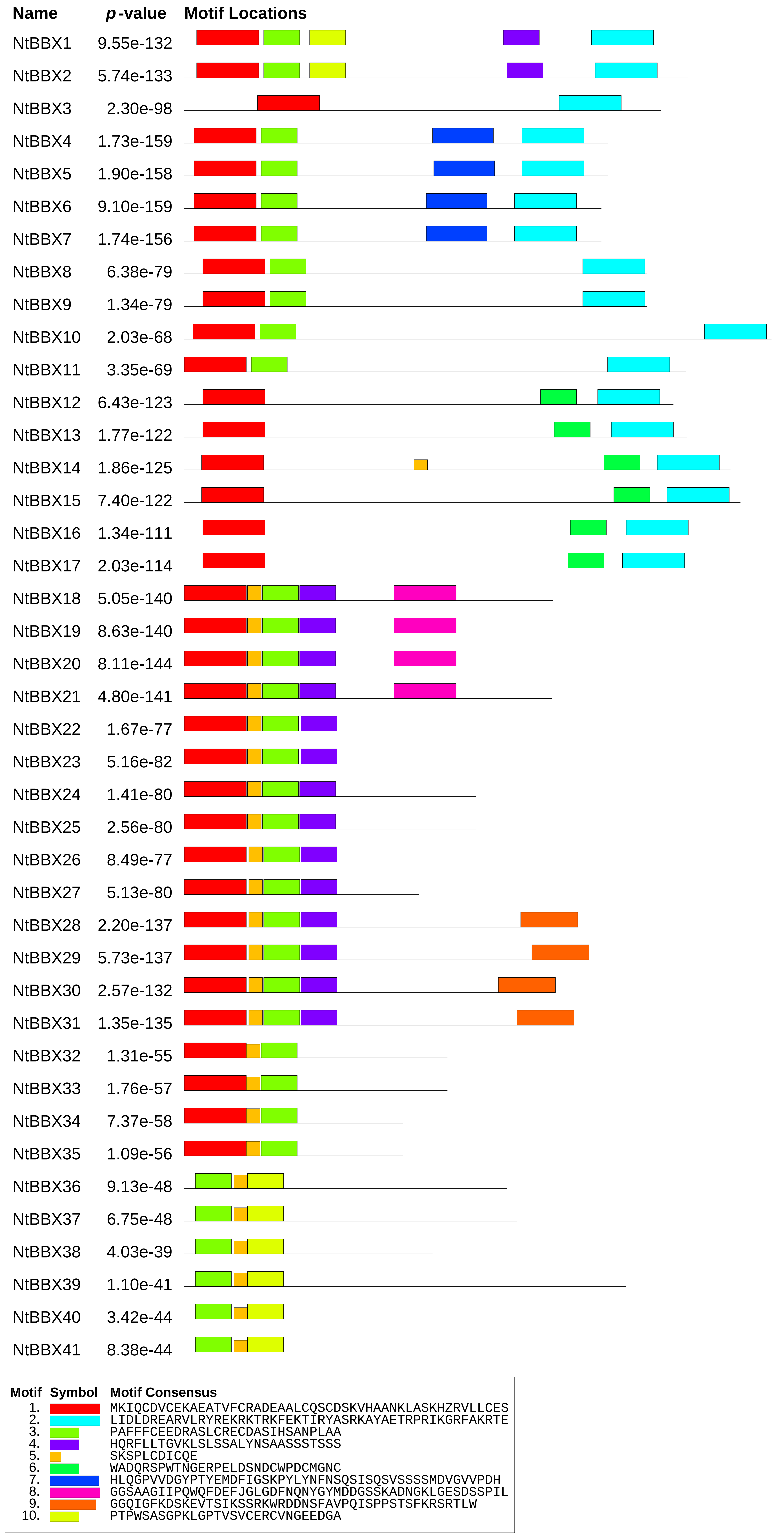
Name
p-value
Motif Locations
NtBBX1 9.55e-132
NtBBX2 5.74e-133
NtBBX3 2.30e-98
NtBBX4 1.73e-159
NtBBX5 1.90e-158
NtBBX6 9.10e-159
NtBBX7 1.74e-156
NtBBX8 6.38e-79
NtBBX9 1.34e-79
NtBBX10 2.03e-68
NtBBX11 3.35e-69
NtBBX12 6.43e-123
NtBBX13 1.77e-122
NtBBX14 1.86e-125
NtBBX15 7.40e-122
NtBBX16 1.34e-111
NtBBX17 2.03e-114
NtBBX18 5.05e-140
NtBBX19 8.63e-140
NtBBX20 8.11e-144
NtBBX21 4.80e-141
NtBBX22 1.67e-77
NtBBX23 5.16e-82
NtBBX24 1.41e-80
NtBBX25 2.56e-80
NtBBX26 8.49e-77
NtBBX27 5.13e-80
NtBBX28 2.20e-137
NtBBX29 5.73e-137
NtBBX30 2.57e-132
NtBBX31 1.35e-135
NtBBX32 1.31e-55
NtBBX33 1.76e-57
NtBBX34 7.37e-58
NtBBX35 1.09e-56
NtBBX36 9.13e-48
NtBBX37 6.75e-48
NtBBX38 4.03e-39
NtBBX39 1.10e-41
NtBBX40 3.42e-44
NtBBX41 8.38e-44
Motif Symbol Motif Consensus
1. MKIQCDVCEKAEATVFCRADEAALCQSCDSKVHAANKLASKHZRVLLCES
2. LIDLDREARVLRYREKRKTRKFEKTIRYASRKAYAETRPRIKGRFAKRTE
3. PAFFFCEEDRASLCRECDASIHSANPLAA
4. HQRFLLTGVKLSLSSALYNSAASSSTSSS
5. SKSPLCDICQE
6. WADQRSPWTNGERPELDSNDCWPDCMGNC
7. HLQGPVVDGYPTYEMDFIGSKPYLYNFNSQSISQSVSSSSMDVGVVPDH
8. GGSAAGIIPQWQFDEFJGLGDFNQNYGYMDDGSSKADNGKLGESDSSPIL
9. GGQIGFKDSKEVTSIKSSRKWRDDNSFAVPQISPPSTSFKRSRTLW
10. PTPWSASGPKLGPTVSVCERCVNGEEDGA
