# Supplementary material for: The Function of BBX Gene Family under Multiple Stresses in Nicotiana tabacum
Source: Genes (Basel). 2022 Oct 12;13(10):1841. doi: 10.3390/genes13101841 (PMC9602306; doi:10.3390/genes13101841)
Supplement: Supplementary file 1 [file genes-13-01841-s001.zip › Table S1ú║Multiple transcripts for four NtBBX genes.pdf]

**Table S1:** Multiple transcripts for four NtBBX genes		
gene ID	gene name	transcript
gene-LOC107814243	*NtBBX6*	**XM_016639620.1**
	NtBBX6IR	XM_016639619.1
gene-LOC107814991	*NtBBX8*	**XM_016640497.1**
	NtBBX8IR	XM_016640496.1
gene-LOC107818998	*NtBBX25*	**XM_016645077.1**
	NtBBX25IR	XM_016645076.1
gene-LOC107771491	*NtBBX32*	**XM_016590865.1**
	NtBBX32IR1	XM_016590866.1
	NtBBX32IR2	XM_016590867.1
